# Supplementary material for: Validation of Breast Cancer Margins by Tissue Spray Mass Spectrometry
Source: Int J Mol Sci. 2020 Jun 26;21(12):4568. doi: 10.3390/ijms21124568 (PMC7349349; doi:10.3390/ijms21124568)
Supplement: Supplementary file 1 [file ijms-21-04568-s001.pdf]

Validation of breast cancer margins by tissue spray mass spectrometry

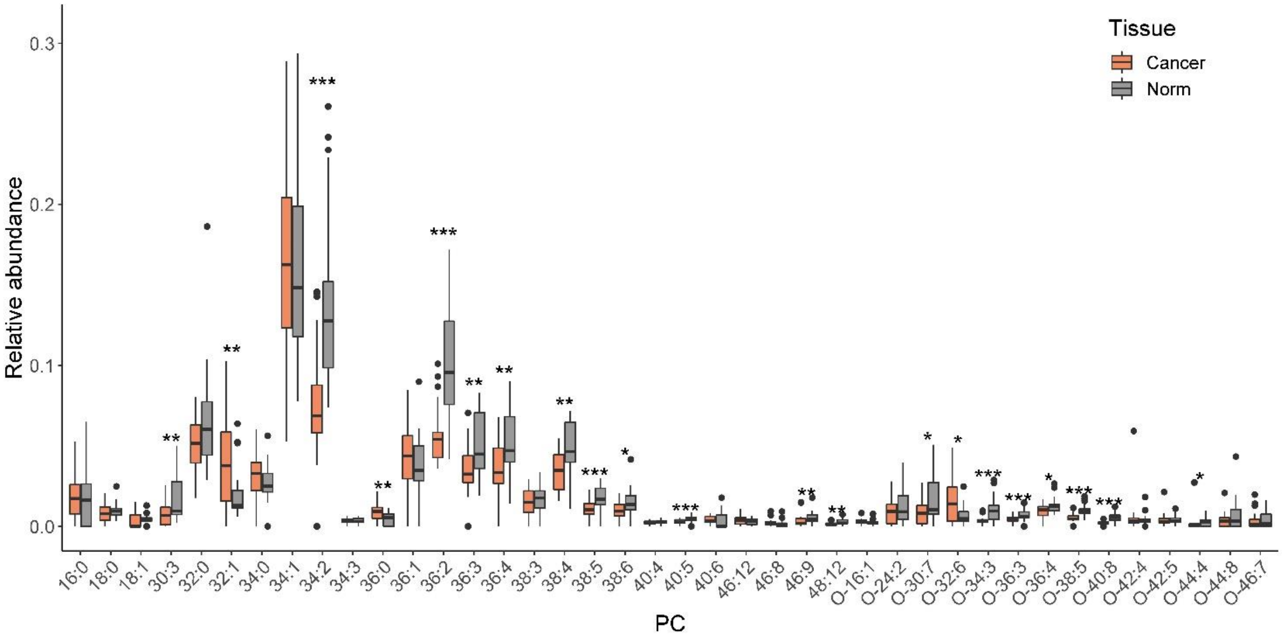

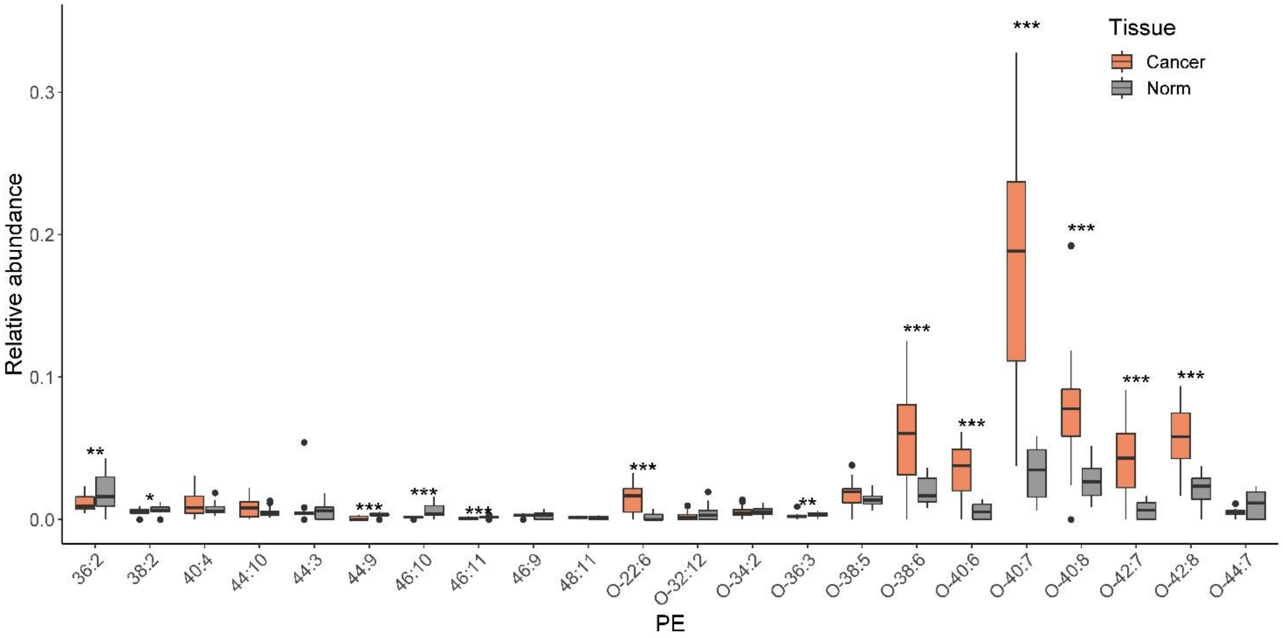

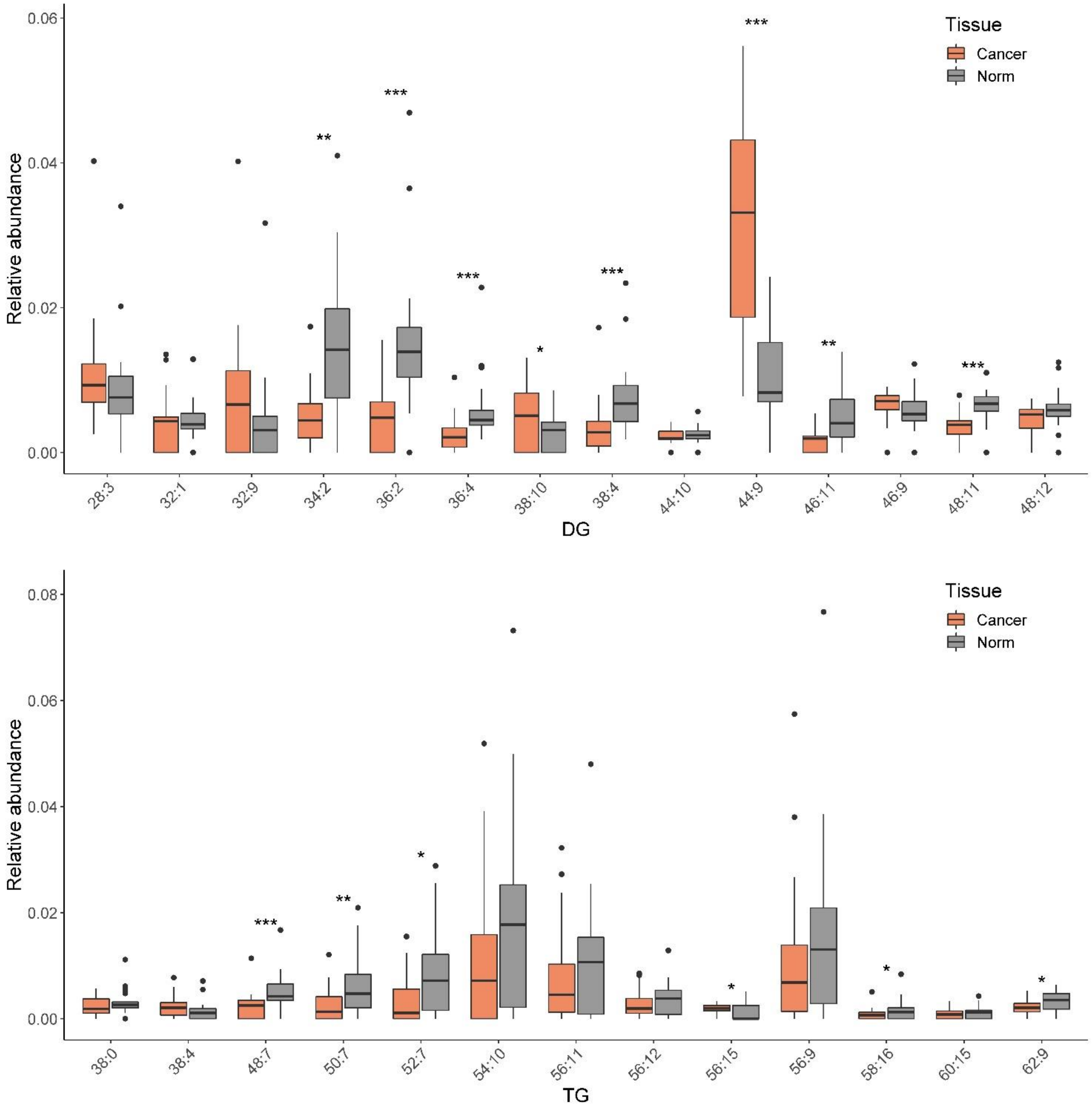

Relative abundance
0.06
0.04
0.02
0.00
Tissue
Cancer
Norm
28:3
32:1
32:9
34:2
36:2
36:4
38:10
38:4
44:10
44:9
46:11
46:9
48:11
48:12
DG
0.08
TG
38:0
38:4
48:7
50:7
52:7
54:10
56:11
56:12
56:15
56:9
58:16
60:15
62:9

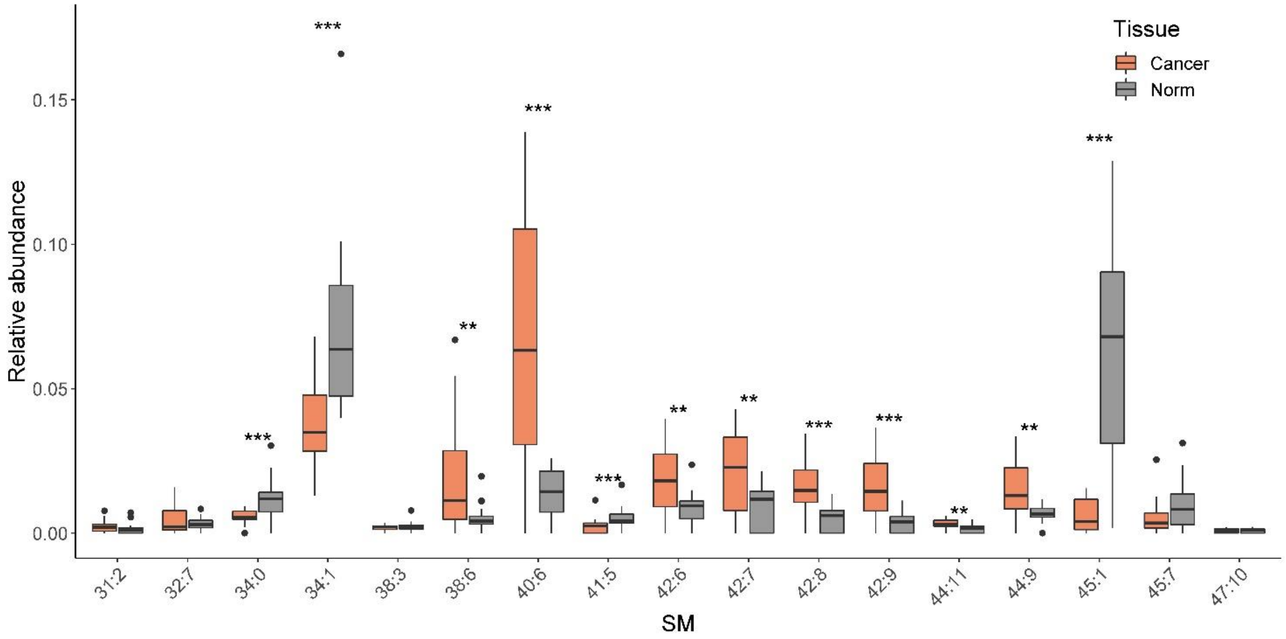

Figure S1. Lipids identified in cancer/ surrounding tissues using tissue-spray MS in positive mode: phosphatidylcholines (PC), phosphatidylethanolamines (PE), diacylglycerols (DG) and triacylglycerols (DG), sphingomyelins (SM). Statistically significant differences according to U-test are indicated by an asterisk: * - p-value < 0.05; ** - p-value < 0.01; *** - p-value < 0.001.

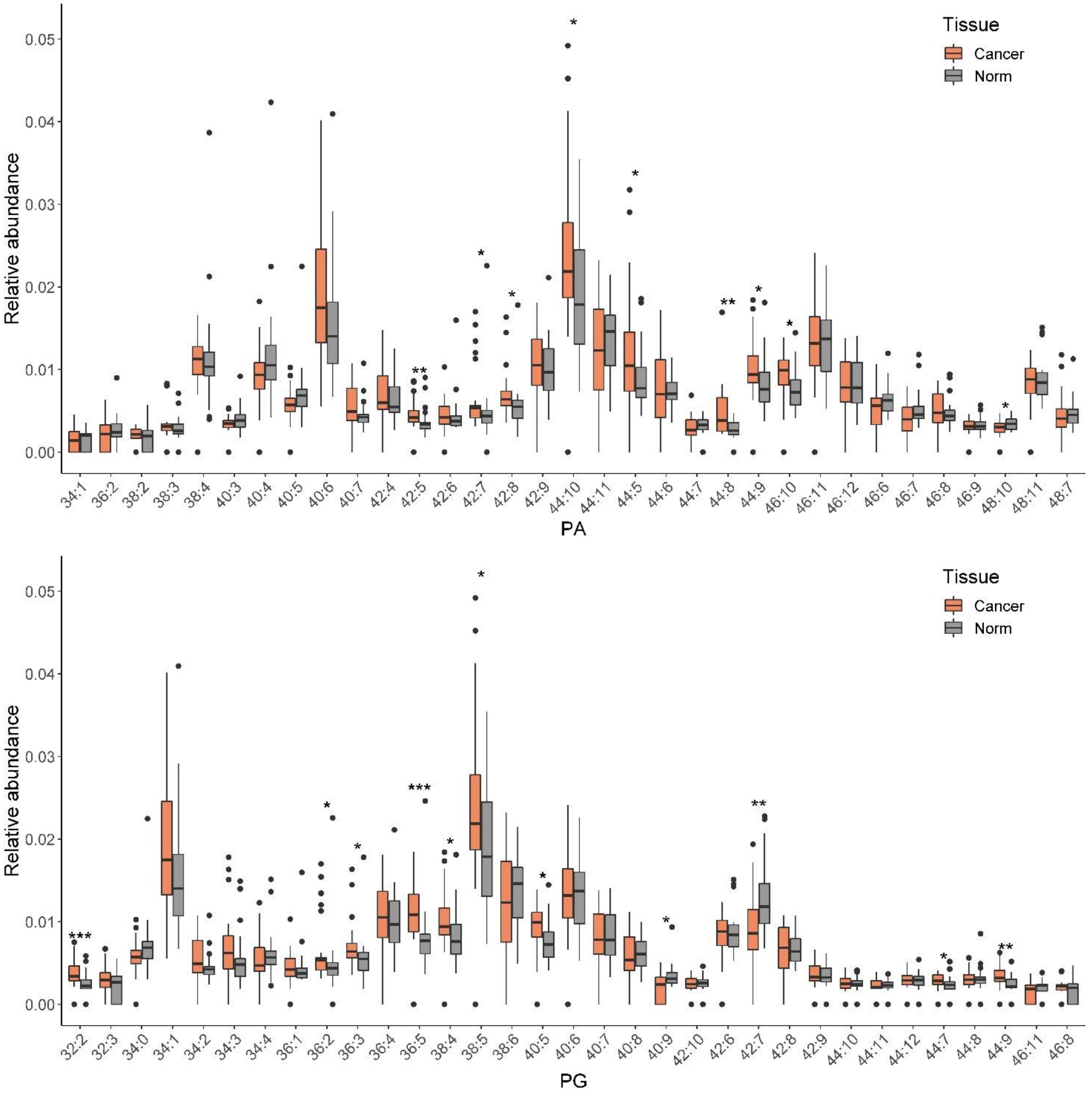

Tissue
Cancer
Norm
Relative abundance
0.00
0.01
0.02
0.03
0.04
0.05
34:1
36:2
38:2
38:3
38:4
40:3
40:4
40:5
40:6
40:7
42:4
42:5
42:6
42:7
42:8
42:9
44:10
44:11
44:5
44:6
44:7
44:8
44:9
46:10
46:11
46:12
46:6
46:7
46:8
46:9
48:10
48:11
48:7
PA
Tissue
Cancer
Norm
Relative abundance
0.00
0.01
0.02
0.03
0.04
0.05
32:2
32:3
34:0
34:1
34:2
34:3
34:4
36:1
36:2
36:3
36:4
36:5
38:4
38:5
38:6
40:5
40:6
40:7
40:8
40:9
42:10
42:6
42:7
42:8
42:9
44:10
44:11
44:12
44:7
44:8
44:9
46:11
46:8
PG

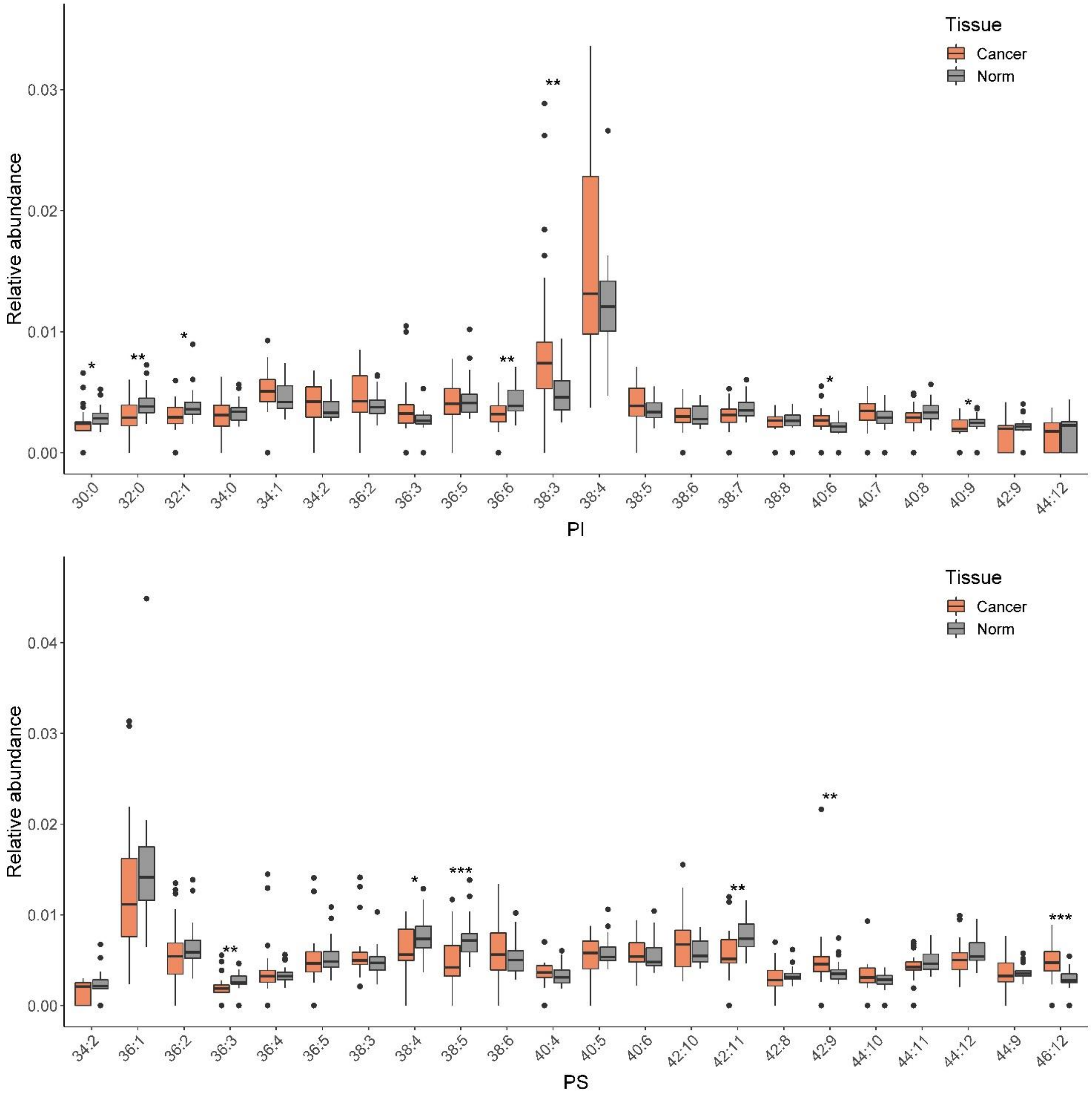

Figure S2. Lipids identified in cancer/ surrounding tissues using tissue-spray MS in negative mode: phosphatidic acids (PA), phosphatidylglycerols (PG), phosphatidylinositols (PI), phosphatidylserines (PS). Statistically significant differences according to U-test are indicated by an asterisk: * - p-value < 0.05; ** - p-value < 0.01; *** - p-value < 0.001.

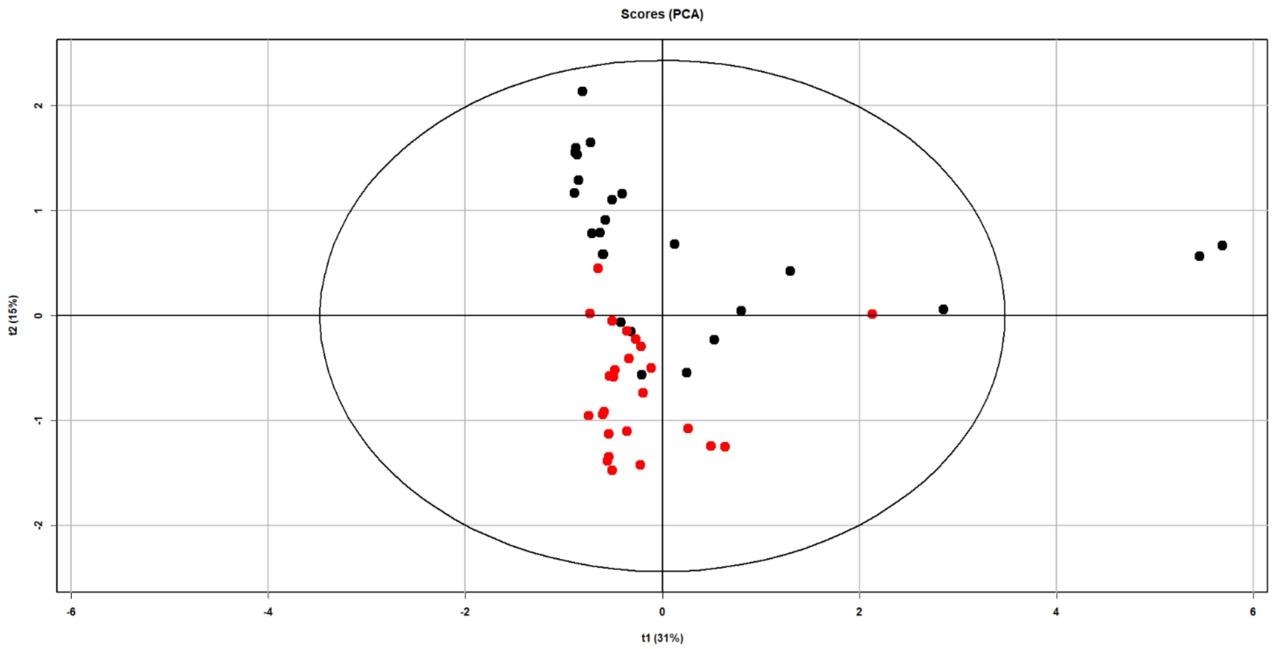

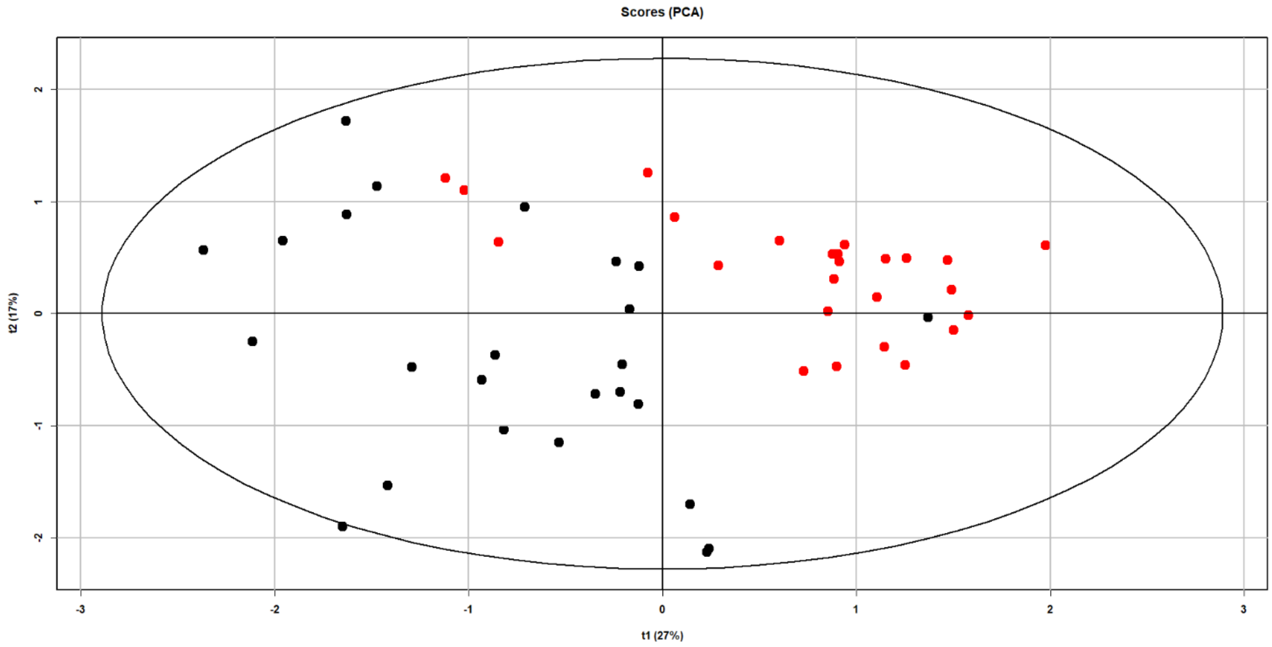

Figure S3. PCA score plots of positive and negative MS.

Table S1. Lipids identified in positive ion tissue-spray mass spectra of breast samples.

Lipid Name	Calculated m/z	Experimental m/z	Error, ppm
DG 28:3	529.3863	529.3912	9
DG 32:1	589.4802	589.4802	0
DG 32:9	573.3550	573.3618	12
DG 34:2	615.4959	615.5004	7
DG 36:2	643.5272	643.5256	2
DG 36:4	639.4959	639.4921	6
DG 38:10	655.4333	655.4241	14
DG 38:4	667.5272	667.5349	12
DG 44:10	739.5272	739.5371	13
DG 44:9	741.5428	741.5479	7
DG 46:11	765.5428	765.5460	4
DG 46:9	769.5741	769.5696	6
DG 48:11	793.5741	793.5701	5
DG 48:12	791.5585	791.5695	14
LPC 16:0	518.3217	518.3271	10
LPC 18:0	546.3530	546.3568	7
LPC 18:1	544.3374	544.3435	11
LPC O-16:1	502.3268	502.3226	8
LPC O-24:2	612.4363	612.4373	2
LPE O-22:6	534.2955	534.3016	11
PC 30:3	722.4731	722.4791	8
PC 32:0	756.5514	756.5557	6
PC 32:1	754.5357	754.5424	9
PC 34:0	784.5827	784.5852	3
PC 34:1	782.5670	782.5728	7
PC 34:2	780.5514	780.5574	8
PC 34:3	778.5357	778.5454	12
PC 36:0	812.6140	812.6046	12
PC 36:1	810.5983	810.6020	5
PC 36:2	808.5827	808.5880	7
PC 36:3	806.5670	806.5710	5
PC 36:4	804.5514	804.5560	6
PC 38:3	834.5983	834.6010	3
PC 38:4	832.5827	832.5864	4
PC 38:5	830.5670	830.5706	4
PC 38:6	828.5514	828.5623	13
PC 40:4	860.6140	860.6205	8
PC 40:5	858.5983	858.6005	3
PC 40:6	856.5827	856.5828	0
PC 46:12	928.5827	928.5888	7

PC 46:8	936.6453	936.6396	6
PC 46:9	934.6296	934.6313	2
PC 48:12	956.6140	956.6258	12
PC O-30:7	700.4313	700.4382	10
PC O-32:6	730.4782	730.4858	10
PC O-34:3	764.5565	764.5455	14
PC O-36:3	792.5878	792.5826	7
PC O-36:4	790.5721	790.5694	3
PC O-38:5	816.5878	816.5940	8
PC O-40:8	838.5721	838.5631	11
PC O-42:4	874.6660	874.6584	9
PC O-42:5	872.6504	872.6433	8
PC O-44:4	902.6973	902.7042	8
PC O-44:8	894.6347	894.6323	3
PC O-46:7	924.6817	924.6868	6
PE 36:2	766.5357	766.5370	2
PE 38:2	794.5670	794.5752	10
PE 40:4	818.5670	818.5565	13
PE 44:10	862.5357	862.5353	1
PE 44:3	876.6453	876.6390	7
PE 44:9	864.5514	864.5392	14
PE 46:10	890.5670	890.5547	14
PE 46:11	888.5514	888.5537	3
PE 46:9	892.5827	892.5753	8
PE 48:11	916.5827	916.5812	2
PE O-32:12	676.3374	676.3317	8
PE O-34:2	724.5252	724.5292	6
PE O-36:3	750.5408	750.5472	8
PE O-38:5	774.5408	774.5316	12
PE O-38:6	772.5252	772.5364	15
PE O-40:6	800.5565	800.5570	1
PE O-40:7	798.5408	798.5459	6
PE O-40:8	796.5252	796.5345	12
PE O-42:7	826.5721	826.5815	11
PE O-42:8	824.5565	824.5558	1
PE O-44:7	854.6034	854.5914	14
SM 31:2	681.4942	681.5012	10
SM 32:7	685.4316	685.4234	12
SM 34:0	727.5724	727.5634	12
SM 34:1	725.5568	725.5611	6
SM 38:3	777.5881	777.5899	2
SM 38:6	771.5411	771.5391	3
SM 40:6	799.5724	799.5635	11

SM 41:5	815.6037	815.6088	6
SM 42:6	827.6037	827.6073	4
SM 42:7	825.5881	825.5807	9
SM 42:8	823.5724	823.5633	11
SM 42:9	821.5568	821.5610	5
SM 44:11	845.5568	845.5557	1
SM 44:9	849.5881	849.5927	5
SM 45:1	879.7289	879.7186	12
SM 45:7	867.6350	867.6341	1
SM 47:10	889.6194	889.6076	13
TG 38:0	689.5691	689.5768	11
TG 38:4	681.5065	681.5012	8
TG 48:7	815.6160	815.6088	9
TG 50:7	843.6473	843.6499	3
TG 52:7	871.6786	871.6900	13
TG 54:10	893.6630	893.6685	6
TG 56:11	919.6786	919.6858	8
TG 56:12	917.6630	917.6697	7
TG 56:15	911.6160	911.6099	7
TG 56:9	923.7099	923.7148	5
TG 58:16	937.6317	937.6273	5
TG 60:15	967.6786	967.6663	13
TG 62:9	1007.8038	1007.7893	14

Table S2. Lipids identified in negative ion tissue-spray mass spectra of breast samples.

Lipid Name	Calculated m/z	Experimental m/z	Error, ppm
FA 16:0	255.2330	255.2296	13
FA 16:1	253.2173	253.2151	9
FA 18:0	283.2643	283.2611	11
FA 18:1	281.2486	281.2455	11
FA 20:2	307.2643	307.2616	9
FA 20:3	305.2486	305.2456	10
FA 20:5	301.2173	301.2152	7
FA 22:4	331.2643	331.2615	8
FA 22:5	329.2486	329.2451	11
FA 22:6	327.2330	327.2304	8
LPE O-16:1	436.2833	436.2802	7
LPE O-26:2	574.4242	574.4189	9
PA 34:1	673.4814	673.4750	10
PA 38:3	725.5127	725.5031	13
PA 40:3	753.5440	753.5380	8
PA 40:6	747.4970	747.4889	11
PA 40:7	745.4814	745.4727	12
PA 42:4	779.5596	779.5536	8
PA 42:5	777.5440	777.5349	12
PA 42:9	769.4814	769.4752	8
PA 44:11	793.4814	793.4711	13
PA 44:5	805.5753	805.5685	8
PA 44:6	803.5596	803.5519	10
PA 44:7	801.5440	801.5357	10
PA 44:8	799.5283	799.5185	12
PA 46:6	831.5909	831.5824	10
PA 46:7	829.5753	829.5650	12
PA 46:8	827.5596	827.5506	11
PA 46:9	825.5440	825.5355	10
PA 48:10	851.5596	851.5498	12
PA 48:11	849.5440	849.5372	8
PA 48:7	857.6066	857.5950	13
PA O-32:3	627.4395	627.4332	10
PA O-36:2	685.5178	685.5113	9
PA O-38:4	709.5178	709.5082	13
PA O-40:5	735.5334	735.5236	13
PA O-44:9	783.5334	783.5239	12
PE 36:2	742.5392	742.5312	11
PE 36:3	740.5236	740.5162	10
PE 38:4	766.5392	766.5317	10

PE 38:5	764.5236	764.5177	8
PE 38:7	760.4923	760.4855	9
PE 40:6	790.5392	790.5296	12
PE 42:8	814.5392	814.5300	11
PE 46:11	864.5549	864.5459	10
PE 46:12	862.5392	862.5279	13
PE 48:12	890.5705	890.5611	11
PE O-34:3	698.5130	698.5043	13
PE O-36:3	726.5443	726.5367	11
PE O-36:5	722.5130	722.5040	13
PE O-36:8	716.4661	716.4567	13
PE O-36:9	714.4504	714.4405	14
PE O-38:6	748.5287	748.5219	9
PE O-38:7	746.5130	746.5041	12
PE O-40:7	774.5443	774.5345	13
PE O-40:8	772.5287	772.5219	9
PE O-44:10	824.5600	824.5493	13
PG 32:2	717.4712	717.4636	11
PG 32:3	715.4556	715.4501	8
PG 34:0	749.5338	749.5239	13
PG 34:3	743.4869	743.4775	13
PG 34:4	741.4712	741.4653	8
PG 36:1	775.5495	775.5439	7
PG 36:2	773.5338	773.5279	8
PG 36:3	771.5182	771.5124	7
PG 36:5	767.4869	767.4763	14
PG 38:4	797.5338	797.5252	11
PG 40:9	815.4869	815.4789	10
PG 42:7	847.5495	847.5377	14
PG 44:10	869.5338	869.5227	13
PG 44:11	867.5182	867.5065	13
PG 44:7	875.5808	875.5737	8
PG 44:8	873.5651	873.5562	10
PG 46:11	895.5495	895.5373	14
PG 46:8	901.5964	901.5875	10
PG O-32:4	699.4606	699.4513	13
PG O-34:4	727.4919	727.4844	10
PG O-34:6	723.4606	723.4515	13
PG O-36:6	751.4919	751.4833	12
PG O-44:11	853.5389	853.5308	9
PI 30:0	781.4867	781.4772	12
PI 32:0	809.5180	809.5087	12
PI 32:1	807.5024	807.4921	13

PI 34:0	837.5493	837.5388	13
PI 34:1	835.5337	835.5252	10
PI 34:2	833.5180	833.5106	9
PI 36:2	861.5493	861.5425	8
PI 36:5	855.5024	855.4939	10
PI 38:3	887.5650	887.5563	10
PI 38:4	885.5493	885.5373	14
PI 38:8	877.4867	877.4806	7
PI 42:9	931.5337	931.5251	9
PS 36:1	788.5447	788.5363	11
PS 36:2	786.5291	786.5207	11
PS 36:3	784.5134	784.5039	12
PS 36:5	780.4821	780.4739	10
PS 38:3	812.5447	812.5336	14
PS 38:5	808.5134	808.5023	14
PS 38:6	806.4978	806.4881	12
PS 40:4	838.5604	838.5491	13
PS 40:5	836.5447	836.5385	7
PS 40:6	834.5291	834.5216	9
PS 42:10	854.4978	854.4859	14
PS 42:9	856.5134	856.5030	12
PS 44:9	884.5447	884.5379	8
PS O-34:5	738.4715	738.4628	12
PS O-36:3	770.5341	770.5256	11
PS O-38:4	796.5498	796.5387	14
PS O-38:5	794.5341	794.5280	8
PS O-40:5	822.5654	822.5546	13
PS O-40:6	820.5498	820.5384	14
PS O-40:7	818.5341	818.5233	13
PS O-42:12	836.4872	836.4805	8
PS O-42:7	846.5654	846.5588	8
PS O-42:8	844.5498	844.5436	7
PS O-42:9	842.5341	842.5276	8
PS O-44:8	872.5811	872.5697	13
PS O-44:9	870.5654	870.5580	9
